# Genetic Diversity of G3 Rotavirus Strains Circulating in Argentina during 1998–2012 Assessed by Full Genome Analyses

**DOI:** 10.1371/journal.pone.0110341

**Published:** 2014-10-22

**Authors:** Juan Ignacio Degiuseppe, Gabriel Ignacio Parra, Juan Andrés Stupka

**Affiliations:** 1 Laboratorio de Gastroenteritis Virales, Instituto Nacional de Enfermedades Infecciosas, Administración Nacional de Laboratorios e Institutos de Salud (ANLIS) “Dr. Carlos G. Malbrán”, Buenos Aires, Argentina; 2 Instituto de Investigaciones en Ciencias de la Salud, Universidad Nacional de Asunción, Asunción, Paraguay; Children's Medical Research Institute, Australia

## Abstract

Seasonal shifts in the predominant strains and the periodic emergence of new strains are epidemiological features of human rotaviruses. After the sporadic detection in two samples in 1998, G3P[8] strains reemerged as the predominant rotavirus during 2008–2009 in Argentina. Notably, in 2011 6.3% (37/587) of samples presented the G3P[6] genotypes, which coincided with the recent detection of G3P[6] and G2P[6] strains in South America and Europe. Analyses of the 11 gene segments of four G3P[8] and two G3P[6] strains revealed that G3P[8] strains detected a decade apart (1998 and 2009) presented minor differences, while the G3P[6] strains presented a complete different genomic constellation albeit showing a similar VP7 gene. This study provides insights in the dynamics and evolution of one of the genotypes with the wider range of hosts and inter-species transmission potential.

## Introduction

Group A rotaviruses are the leading cause of sporadic acute diarrhea in young children worldwide. It is estimated that rotaviruses are associated with over 450,000 deaths in that age group, most of them in developing countries [1].

Rotavirus genome comprises 11 segments of double-stranded RNA (dsRNA), which are surrounded by a triple layered capsid protein. The outermost proteins of the capsid (VP7 and VP4) are responsible for eliciting neutralizing antibodies, and therefore the major targets for vaccine development. Genetic and antigenic differences within the VP7 and VP4 have been used to classify rotavirus into G and P types, respectively [2]. Despite that multiple genotypes (27 G types and 37 P types) have been described [3]–[4], only few of them are predominant in humans (i.e. G1P[8], G2P[4], G3P[8], G4P[8], and G9P[8]) [5]. Seasonal changes in the predominant strain and the periodic emergence of uncommon strains (like G12 or G8), are hallmarks of rotavirus epidemiology [5].

Rotaviruses evolve through multiple genetic mechanisms, such as accumulation of point mutations, reassortment, and intragenic recombination [6]–[10]. The analysis of the 11 genome segments has shown to be a useful tool to assess the evolutionary relationships among different strains, track reassortment events, and understand the origins of uncommon rotavirus [11]. Based on genetic analyses, each genome segment has been classified into multiple genotypes, and large-genome analyses revealed the association of genotypes that are known as genome constellations [11]–[12].

Our laboratory has been leading the National Rotavirus Strain Surveillance Network from Argentina, and rotavirus-positive samples from different geographical regions of the country have been characterized since 1996. Overall, G1P[8] rotaviruses were the most frequently detected in Argentina, followed by G4P[8], G2P[4]-which showed a cyclic pattern of predominance every 7 years-, and G9P[8] -which emerged in 2006- [13]–[17]. G3P[8] is one of the most common rotavirus strains in humans worldwide; however, in Argentina were sporadically detected (2/1764, 0.11%) from 1997–2007 [14]. In 2008, an increased frequency of detection of G3P[8] strains was observed reaching up to 54.1% of the strains analyzed in 2009 [18]. Interestingly, in 2011 the genotype G3P[6] was first detected in Argentina in 37 samples (6.3%) [19]. This finding coincided with the recent detection of G3P[6] and G2P[6] strains in Brazil and Belgium [20]–[21].

Two different rotavirus vaccines, Rotarix and RotaTeq, are being use for children immunization in different countries [22]–[23]. These vaccines have been licensed in 2006 in Argentina, but until now they were not included into the National Immunization Program. Thus, their use is restricted to the private health sector, and estimations have shown that only 10% of the live birth cohort received the vaccine. Efficacy of vaccines depends in multiple factors, such as malnutrition, presence of maternal antibodies and the circulating strains [24]–[25]. Thus, robust surveillance systems and the understanding of rotavirus evolution will help us to evaluate the future vaccination programs. To gain insights into the genomic characteristics and evolution of G3 strains detected in Argentina, we sequenced the 11 gene segments of four G3P[8] and two G3P[6] strains, which revealed that G3P[8] strains from a decade apart (1998 and 2009) presented minor differences, while the G3P[6] strain that emerged in 2011 presented a complete different genomic constellation, albeit showing a similar VP7 gene.

## Materials and Methods

### Sample collection and G and P typing

Rotavirus-positive samples were detected by ELISA or immunochromatography by Sentinel Units across the country, and sent to the Rotavirus National Reference Laboratory for further genotype characterization. G and P typing was performed by reverse-transcriptase polymerase chain reaction (RT-PCR) followed by nested-multiplex PCR with consensus and type-specific primers [26]–[28].

### Complete genome characterization

To analyze the diversity of G3 strains, nearly the full length (>95%) of their genome was sequenced in six strains: 2 G3P[8] from 1998 (Arg1240 and Arg1759), 2 G3P[8] from 2009 (Arg6795 and Arg7338) and 2 G3P[6] from 2011 (Arg9448 and Arg9467). The two G3P[8]-2009 and two G3P[6]-2011 strains were randomly selected from a much larger collection of G3P[8] (n = 720) and G3P[6] (n = 37) strains circulating during 2009–2011. In over 50% of this collection, RNA migration in polyacrilamide gel electrophoresis (PAGE) was performed. All the G3P[8] strains presented the same long electropherotype, and all the G3P[6] presented the same short electropherotype (data not shown). Thus, high similarity for strains bearing the same G and P association was assumed.

Viral dsRNA was extracted using automated QIAcube protocols (Qiagen, CA, USA), and the 11 segments were amplified by RT-PCR using Qiagen One Step RT-PCR kit (Qiagen, CA, USA) according to the manufacturer's instructions. PCR products were purified with a QIAQuick PCR purification kit (Qiagen, CA, USA) and the sequencing was performed using the dideoxynucleotide chain termination method with the BigDye Terminator v3.1 Cycle Sequencing kit (Applied Biosystems, CA, USA) on a 3500Dx Genetic Analyzer automated sequencer (Applied Biosystems, CA, USA). Primers targeting the conserved 5′- and 3′-end regions as well as internal primers were used for RT-PCR and sequence reactions (primer sequences are available upon request). All gene contigs were assembled with a coverage of 2× or more. The nucleotide sequences have been deposited in GenBank under accession numbers KJ583136-KJ583157 (Arg1240 and Arg1759), KJ583158-KJ583179 (Arg6795 and Arg7338) and KJ583180-KJ583201 (Arg9448 and Arg9467).

### Sequence and phylogenetic analyses

The analyses were conducted on the G3 strains reported in this work, as well as different strains in which the eleven genes sequences were available in GenBank. The alignments were carried out using BioEdit v7.0.1 [29]. Nucleotide and amino acid differences were calculated using p-distance model, and the phylogenetic trees were reconstructed for each of the 11 gene segments using the neighbor-joining method, Tamura-Nei (TN93) as substitution model and gamma-distributed rate variation among sites as implemented in MEGA version 6 [30].

## Results

### Genomic Constellation

**Figure 1:**
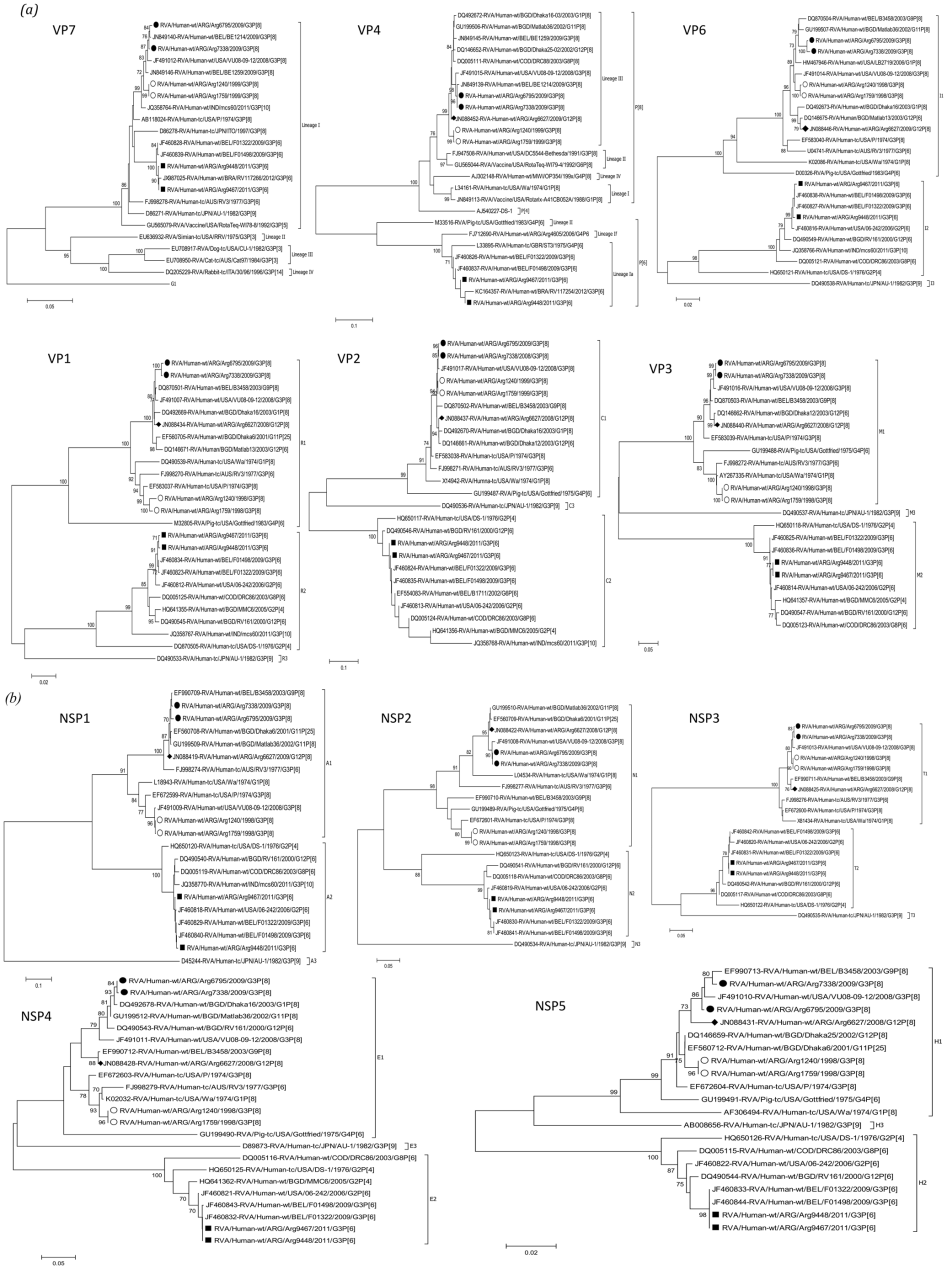
Phylogenetic analysis and genotype assessment. Phylogenetic trees of (*a*) viral proteins and (*b*) non-structural protein coding gene segments of relevant rotavirus strains used for comparison with the Argentinean G3P[8] and G3P[6] strains. Trees were calculated with the neighbour-joining method, Tamura-Nei substitution model (TN93) and gamma-distributed rate variation among sites. Bootstrap values were calculated using 1000 replicates (values >70% are shown). The Argentinean G3P[8] strains detected in 1998 are marked with an empty circle, the G3P[8] strains detected in 2008/9 with filled circle, the Argentinean G3P[6] strains with filled square, and an Argentinean G12P[8] strain with a diamond.

**Figure 2:**
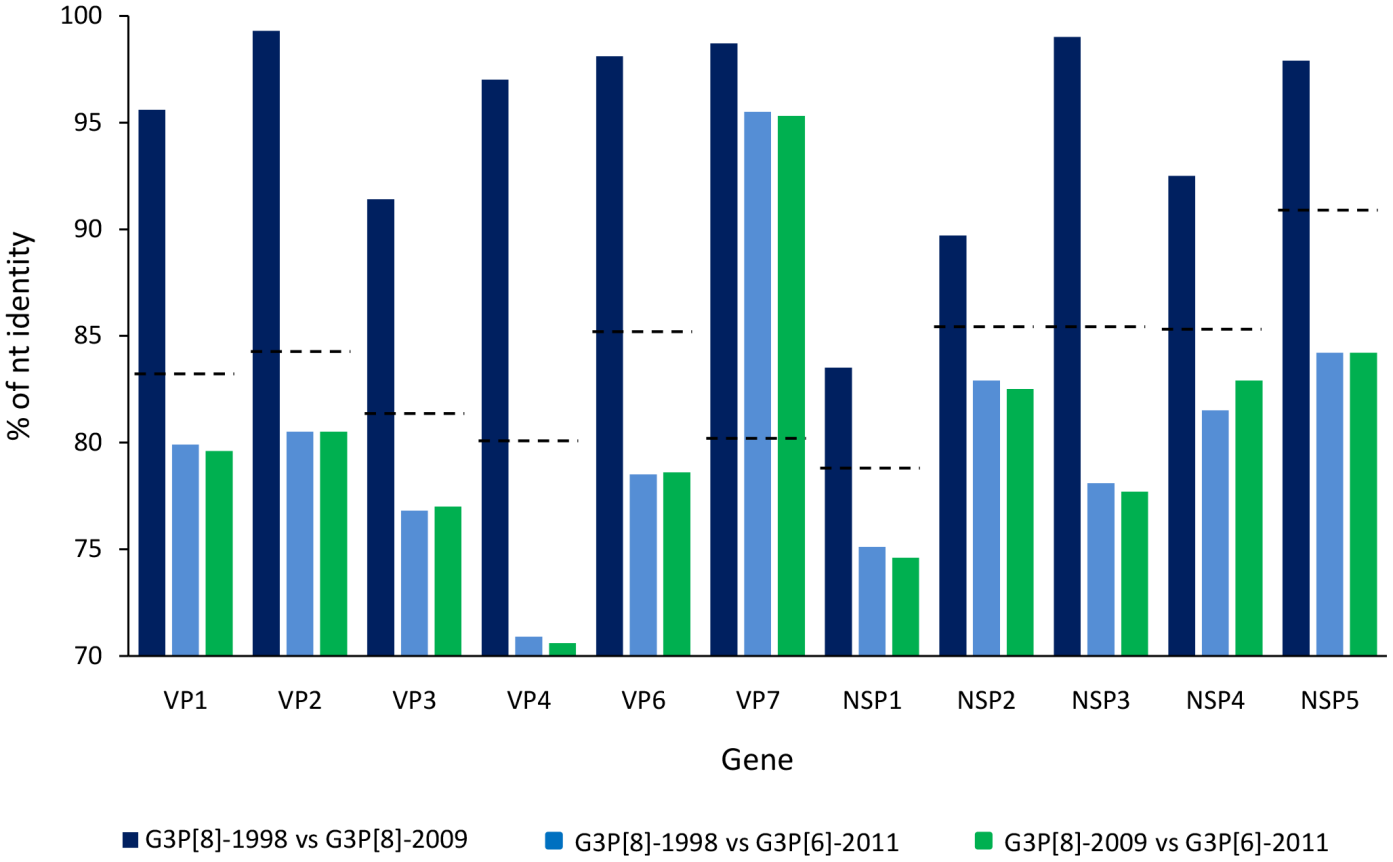
Genetic differences among Argentinean G3 strains. Nucleotide identities among the G3 Argentinean strains belonging to the three studied seasons are shown in different bars. Cut-off values for genotype assignment are indicated for each gene with dash lines.

Sequencing of two representative G3 strains from three different seasons (1998, 2009 and 2011) was conducted. Overall, G3 strains detected in the same season were nearly identical between them (>99% at nucleotide level). Phylogenetic analyses of the four G3P[8] strains showed a Wa-like (I1-R1-C1-M1-A1-N1-T1-E1-H1) genome constellation, while G3P[6] strains presented a DS1-like (I2-R2-C2-M2-A2-N2-T2-E2-H2) backbone (Fig. 1). Analyses of the nucleotide identity among the G3 strains from the three seasons confirm the genetic differences among the G3P[8] and G3P[6] strains in all genes but VP7 (Fig. 2).

### Comparison of Argentinean G3 strains

**Figure 3:**
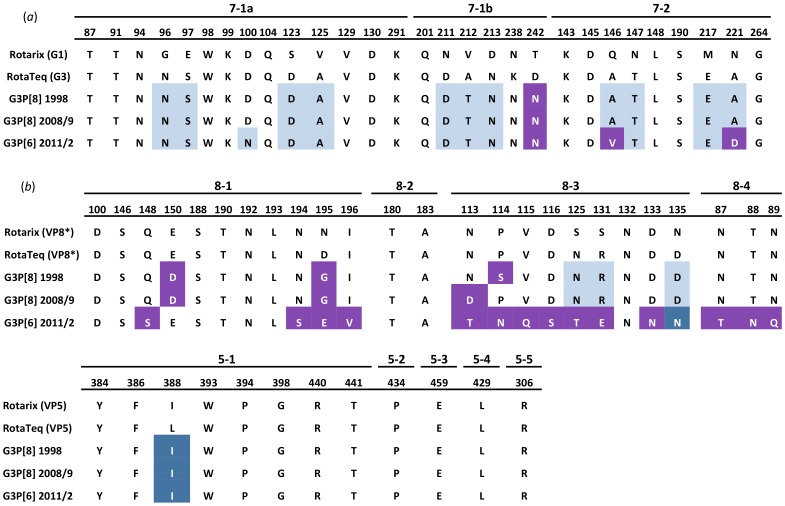
Differences in antigenic sites among the Argentinean G3 strains and the vaccine strains. Amino acid residues in VP7 (a) and VP4 (b) antigenic sites are shown for the three different Argentinean strains and vaccine strains.

Phylogenetic analyses of the VP7 gene clustered all Argentinean G3 strains within the major lineage of human G3 (lineage I), and all the VP7 genes showed 95.3% and 96.2% of similarity at nucleotide and amino acid level, respectively. Examination of individual amino acid changes in the VP7 revealed that 11 changes were observed between G3P[8] strains and the G3P[6] strains, 3 of them located at antigenic sites (D100N, from antigenic region 7–1a and A146V and A221D, from antigenic region 7–2) (Fig. 3a). With regards to the VP4 gene, the G3P[8] strains grouped in P[8]-3 lineage (most common lineage in humans), while the P[6] strains grouped in P[6]-1a lineage along with other human P[6] strains (Fig. 1a) [16]. As expected, the VP4 gene segments of the P[8] and P[6] strains showed similarities around 70% at nucleotide level (Fig. 2), and more than 165 amino acid changes along the complete protein. When comparing the G3P[8] strains that were detected a decade apart (i.e. G3P[8]-1998 and G3P[8]-2009 strains), their VP7 showed just one amino acid change in a non antigenic site (Y235H), while their VP4 presented 15 amino acid changes, two of them (N113D and S114P) located in a protective antigenic site (8–3) (Fig. 3a, b). When analyzing the remaining nine genes, despite presenting the same Wa-like constellation, G3P[8]-1998 and G3P[8]-2009 strains grouped in different clusters in 6 of them (Fig. 1). It is noteworthy that for three gene segments (VP1, VP3, and NSP4), all the strains analyzed were grouped by temporal patterns (i.e. strains circulating before and after year 2000). The highest amino acid difference was observed in NSP1, with 80 amino acid changes (18.5%). With the exception of the VP6 and NSP1, the phylogenetic trees revealed that the Argentinean G3P[8]-2009 strains were more closely related to the contemporary G3P[8] strains detected in USA, than the Argentinean G3P[8]-1998 (Fig. 1).

Analyses of the Argentinean G3P[6]-2011 genes showed that despite presenting a typical Genotype 2 constellation, all of them clustered distantly from the prototype strain DS1 (G2P[4]); and more closely related to the G3P[6] and G2P[6] strains recently detected in Belgium and USA [19]–[20], respectively (Fig. 1). The VP7 from South American and European G3P[6] strains shared the same amino acid sequence, except for one residue (D100N) located in the antigenic region 7–1a (Fig. 3). Comparison between Argentinean and Belgian strains showed nearly identical amino acid sequences in the VP4 gene segment, with only two residues changes at position 460 and 581 (VP5 region). Only the VP8* region of the VP4 was available for the Brazilian strains, and they presented the same amino acid sequence among them and the Argentinean and Belgian strains.

### Comparison of Argentinean G3P[8] and G12P[8] strains circulating in the same season

**Figure 4:**
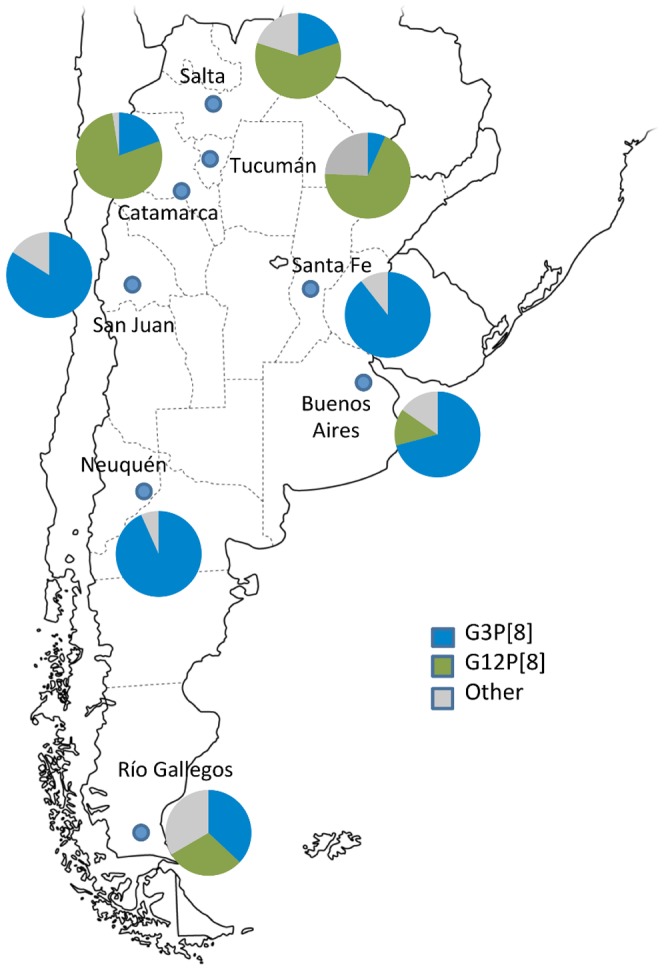
Rotavirus strain distribution in Argentina, 2009. Surveillance for rotavirus strains was conducted in eight cities during 2008–2009 [18]. Frequency of detection for G12P[8], G3P[8] and other strains are shown in pie charts for each city.

During 2008–2009, G12P[8] and G3P[8] strains co-circulated at similar frequencies in Argentina [18]; however, in most of the cities where the survey was conducted one strain predominated over the other one (Fig. 4). We previously shown that the G12P[8] strains presented a Wa-like (Genotype 1) genome constellation [18], and comparison with the G3P[8]-2009 strains showed that they grouped in the same genotype but different clusters in all the 11 genes but VP7 gene (Fig. 1). With the exception of the G type specificity, amino acid comparison showed high degree of similarity (>96.5%) between these two co-circulating strains (data not shown).

### Comparison of Argentinean G3 strains with vaccine strains

When compared the G3 Argentinean strains and the VP7 and VP4 vaccine components, the Argentinean G3P[8] strains were more related (96.9% at amino acid level) to the G3 component (WI78-8) of the RotaTeq vaccine than the G3P[8] strains (94.8% at amino acid level). Three residues from the antigenic region 7–1b were found to differed between the G3P[8] strains and WI78-8: A212T, K238N and D242N. Moreover, G3P[6] strains showed three additional changes at antigenic regions 7–1a (D100N) and 7–2 (A146V and A221D). Higher differences were seen when comparing Argentinean strains with Rotarix (a G1P[8] strain). Twelve changes, four in each of the three VP7 antigenic regions, were observed for G3P[8] strains and thirteen changes were observed for the G3P[6] strains. When analyzing the VP4 gene, Argentinean G3P[8] strains were found to have six amino acid changes compared to Rotarix (all of them located at the VP8* region), and only four differences with RotaTeq (three at VP8* region and one at VP5* region). On the other hand, G3P[6] strains showed 14 amino acid changes with Rotarix (all of them at antigenic sites of VP8* region) and 16 residues changes with RotaTeq (15 of them at the antigenic sites of VP8* region and one at the VP5* region) (Fig. 3a, b).

## Discussion

G3 rotaviruses are considered one of the most frequently detected strains worldwide [5]. This genotype presents the broadest host range (infecting humans, pigs, horses, cows, rabbit, cats, dogs, among others) and, consequently, the highest number of P type associations (i.e. P[2], P[3], P[6], P[7], P[8], P[9], P[12], P[14], P[22], P[24]) described for any other rotavirus G type [31]–[34]. Despite this extreme diversity, a single G and P combination (G3P[8]) is the most prevalent G3 infecting humans. G3P[8] strains have been described circulating at low rates during the 1990s worldwide, but its re-emergence has been widely documented in the last decade [35]–[40]. Its increased frequency in most of the countries is supposed to be at expenses of the decrease of frequencies of G1P[8] and G9P[8] [18], [41]–[42]. In Latin America, G3P[8] has been barely detected (i.e. <0.5%) [43]–[44] until recently, when >39% of the rotavirus strains circulating in Argentina during 2008–2010 presented this G and P combination [18]–[19].

To gain insights into the characteristics that may lead to G3P[8] to prevail in Argentina since 2009, we compared the genomes of G3P[8] shown circulating a decade apart, and with the newly emergent G3P[6] strains. All G3 strains from this study presented very low heterogeneity in the VP7 protein and, despite of being detected 10 years apart, no significant amino acid changes at the VP7 and VP4 proteins were detected. Multiple changes at different antigenic regions were described in the recently emergent G3P[8] strains in different countries of Asia [39]–[40], [45]–[46]. However, the low heterogeneity of VP7 and VP4 after 10 years rules out that major antigenic changes accounted for the re-emergence of G3P[8] strains in Argentina.

When we examined the genomic differences among the Argentinean G3P[8]-1998 and G3P[8]-2009, minor differences can be detected for most of the genes. The major differences were observed in NSP1 gene segment, which is the one implicated in viral antagonism of the interferon response from the host [47]–[48]. Several studies had indicated this protein as the most variable among rotavirus strains (i.e. sequence identities below 40% among mammalian rotaviruses) [47], [49]–[50], and those differences are implicated in the ability of rotavirus to antagonize the innate immune responses of the host [51]. Thus, the differences presented by G3P[8] in the NSP1 may play a role in the better fitness of the 2009 strains. Of note is that in some genes (VP1, VP3 and NSP4), there is a temporal clustering of the strains; where G3 strains circulating after 2000 cluster together regardless of the country of detection. Thus, there seems to be a turning point in the evolution of G3 that lead to the re-emergence after of the low prevalence in the human population during 1990s.

Besides of the VP7 gene, G3P[8]-2009 strains presented the same genomic backbone than the G12P[8] strains, which co-circulated in the same season in Argentina [18]. The fact that both (G3 and G12) strains presented the same genetic backbone and alternated the predominance in different cities during the same season [18], suggest that the strains might be competing for the same susceptible population and one excludes the other one in a given geographical location (Fig. 4). Although, it remains unclear how an emergent strain acquires advantages over others and predominate at a given time and space, possible explanations may reside at multilevel factors ranging from host susceptibility to environmental aspects.

As G3 strains seem to be spreading more efficiently since 2000s, unusual associations are therefore more predisposed to be detected infecting human (i.e. G3P[4] and G3P[10]) [52]–[53]. In Argentina, novel G3P[6] strains were detected at considerable rates in 2011 (i.e. 6.3%), unlike the low prevalence described in a recent Latin American meta-analysis (i.e. 0.2%) [44]. These strains presented, except for the VP7 gene segment, a genotype 2 constellation backbone (DS1-like) of human origin. Recently, G3P[6] strains were reported in Brazil and Belgium [20]–[21]. Comparison between the Argentinean G3P[6] strains and these reported recently in other countries, showed high similarity and clustering in all 11 gene segments. Interestingly, one amino acid difference at the VP7 antigenic region 7-1a was observed between South American and European strains. This finding reflects the efficiency and speed by which rotaviruses evolve for optimal regional spreading. Although G3P[6] strains presented genotype 2 constellation, they were found to be more closely related to strains bearing P[6] genotype than to DS-1 prototype strain [20]. This fact reinforces the evidence of a probable Asian/African origin with a rapid spread through the world, rather than being an intergenotypic reassortant originated from two independent gene-exchanging steps.

Argentinean G3P[8] strains showed fewer amino acid changes than G3P[6] strains with the RotaTeq G3 vaccine component. When compared the VP4 antigenic sites, P[8] strains were found to be more closely related to RotaTeq than Rotarix. The fact that G3P[8] strains are more similar to vaccine components than G3P[6] strains seems to represent a challenge to current vaccines for the latter strain because of the higher number of differences at antigenic sites, i.e. a P[6] component with a DS-1 like backbone. Because coverage of vaccine do not exceeds 10% of Argentinean live birth cohort, the emergence and variation of strains within our country seems to reflect a natural fluctuation of rotavirus; however, we cannot rule out that massive vaccination in neighboring countries could play a role in emergence of new strains. Rotaviruses constantly show us unpredicted scenarios; therefore robust and continuous epidemiological surveillance must be conducted with the goal of understanding rotavirus dynamics and evolution.
